# A case report of primary meningococcal pericarditis secondary to *Neisseria meningitidis* in a young female patient

**DOI:** 10.1016/j.idcr.2022.e01634

**Published:** 2022-11-01

**Authors:** Matthew Green, Peter Harrison, Anshuman Sengupta, Dominik Schlosshan

**Affiliations:** Leeds General Infirmary, Leeds Teaching Hospitals NHS Trust, Great George Street, Leeds LS1 3EX, United Kingdom

**Keywords:** Pericarditis, Pericardial effusion, Meningococcus

## Abstract

Pericarditis is responsible for approximately 5 % of emergency admissions due to chest pain. Pericarditis secondary to Neisseria meningitidis (meningococci) was originally reported in 1918, and remains a rare diagnosis. We report a case of primary meningococcal pericarditis presenting with non-specific symptoms, illustrating the importance of considering rarer causes of pericardial effusion. A previously fit and well 23-year-old female presented to her local hospital with a 2-day history of feeling generally unwell with myalgia and fevers and was initially discharged. Four days following discharge the patient re-presented with worsening symptoms. A Computed Tomography Pulmonary Angiogram (CTPA) demonstrated a large pericardial effusion with subsequent bedside echocardiogram confirming a global pericardial effusion of up to 3 cm. This required drainage, with blood cultures and pericardial fluid showing polymerase chain reaction positivity for Neisseria meningitidis, serogroup B. Our report describes a rare case of Primary Meningococcal Pericarditis secondary to serotype B meningococcal infection. The European Society of Cardiology propose criteria that warrant hospital admission and an aetiology search for certain patients with pericardial disease. These criteria provide a useful framework to help select those minority of patients in whom a more serious underlying cause is present. Blood cultures provide vital information to allow us to complete a thorough aetiological search and empirical antibiotics can cloud the clinical picture, making it harder to identify causative organisms. To aid the early administration of appropriate therapy, it may be pertinent to recommend a low threshold for taking blood cultures in patients with pyrexia and pericarditis or pericardial effusion.

## Highlights

• Pericarditis caused by Meningococcus is rare but serious.
• Consider alternative causes of pericarditis, particularly in young people.
• Patients with bacterial pericardial effusions have high mortality and morbidity.
• Early blood cultures are essential for treating rarer forms of pericarditis.

## Introduction

Pericarditis is a relatively common condition, responsible for 0.1% of all hospital admissions and 5% of emergency admissions with chest pain [1]. There are a wide range of aetiological factors, which may be broadly categorised as infective or non-infective. Despite this variation, in developed countries around 80–90 % are considered idiopathic, with the majority of these presumed secondary to viral infection [2]. Pericarditis secondary to bacterial infections represent only a minority of cases, with a reported incidence of < 1 % [3]. Commonly implicated organisms in such cases are: *Staphylococcus spp*, *Streptococcus spp, Haemophilus spp*, and *M. tuberculosis*, with meningococci accounting for approximately 6 % of non-tuberculous bacterial pericarditis cases [4]. Whilst uncommon, bacterial pericarditis is a serious and potentially fatal disease with a high mortality rate of ∼ 40 % in treated patients and 85 % in untreated [5], [6].

Pericarditis secondary to *Neisseria meningitidis* was originally reported in 1918, and may be classified into 3 groups: disseminated meningococcal disease (DMD); primary (or isolated) meningococcal pericarditis (PMP) and immune-reactive (or reactive) meningococcal pericarditis (IRP) [7], [8].

We report a case of primary meningococcal pericarditis presenting with non-specific symptoms, illustrating the importance of considering rarer causes of pericardial effusion.

## Case Report

A previously fit and well 23-year-old female presented to her local hospital with a 2-day history of feeling generally unwell with myalgia and fevers. She was initially discharged following a negative COVID-19 swab, despite a high clinical suspicion for COVID-19 at the time. Four days following discharge the patient re-presented to the same hospital with worsening symptoms, now including dyspnoea, vomiting and chest tightness. On re-presentation she was tachypneic with a respiratory rate of 34, maintaining adequate oxygen saturation of 95 % on room air, tachycardic (110 beats per minute) with a blood pressure of 105/55 mmHg. On examination she was tender in the epigastrium and heart sounds were reported as normal, there was no evidence of peripheral oedema or signs of deep venous thrombosis.

Electrocardiography (ECG) demonstrated concave-upward ST segment elevation in leads I and II with reciprocal changes in V1. Chest X-ray demonstrated cardiomegaly, right sided pleural effusion and left basal consolidation. Inflammatory markers were raised, with a C- reactive protein (CRP) of 241 mg/L and white cell count (WCC) of 11.6. Liver function was also notably deranged with an alanine aminotransferase (ALT) of 695 International Units/Litre (IU/L) and alkaline phosphatase (ALP) of 266 IU/L in the presence of a normal bilirubin. D-dimer was markedly raised at 18.02 mg/L fibrinogen equivalent units. Troponin was normal. Initially a wide range of differential diagnoses were discussed, including pulmonary embolism and acute cholecystitis. Therefore, further imaging with a CT pulmonary angiogram, plus extended abdominal cover was agreed. This ruled out pulmonary embolism but demonstrated a large pericardial effusion and bilateral pleural effusions. Subsequent transthoracic echocardiogram confirmed a global pericardial effusion of up to 3 cm, mildly compromising right ventricular (RV) function, and a dilated inferior vena cava. Left ventricular (LV) function was normal and no valvular abnormalities were demonstrated. Based on these findings the patient was transferred to a tertiary cardiology centre for ongoing management including the consideration of pericardiocentesis. Upon arrival to our centre the patient was symptomatic with dyspnoea and had a sinus tachycardia with pulsus paradoxus. On the basis of these findings and early echocardiographic features of tamponade, she underwent apical pericardiocentesis. 650 mL of straw-coloured fluid were drained and the patient significantly improved symptomatically post-procedure. A pericardial drain was left *in situ*.

A single dose of both ceftriaxone and metronidazole had been given before transfer to cover for acute cholecystitis given the deranged liver biochemistry. Blood cultures and samples of pericardial fluid were sent for analysis at this stage. Clavulinic acid/amoxicillin was commenced to cover the possibility of superadded bacterial pneumonia, with or without an underlying COVID-19 infection, although this was deemed increasingly unlikely in the clinical context and following a further negative COVID-19 swab. Serum Antibodies/Antigens for Hepatitis C and Human Immunodeficiency Virus (HIV) 1 & 2 were not detected. Following subsequent discussion with the microbiology team the antibiotic regimen was switched to piperacillin-tazobactam and clarithromycin.

The following day, repeat echocardiography demonstrated a significant residual global rim of pericardial fluid. At this point a further 100 mL were aspirated. Two days after initial transfer, blood cultures showed polymerase chain reaction (PCR) positivity for *Neisseria meningitidis*, serogroup B, prompting a switch in antibiotic regimen back to intravenous ceftriaxone and metronidazole. A further five days later, pericardial fluid PCR also showed *N. meningitidis.* On review of the patient’s vaccination history it was noted that she had received a single Meningococcal C vaccination in 2000 and the Nimenrix vaccination (containing Meningococcal A, C, W-135 and Y) in 2015, around four years before her acute presentation.

Serial daily echocardiograms demonstrated improvement in the patient’s residual effusion, without further tamponade. The pericardial drain was removed three days after insertion. The patient was discharged 21 days after initial presentation with a short course of colchicine and no further antibiotic therapy. An outpatient review approximately 1 month after discharge demonstrated that she was back to her level of baseline physical fitness. Her repeat ECG was essentially unremarkable and echocardiography demonstrated normal left and right heart size, normal LV function and mildly impaired RV systolic function. No residual fluid was evident and no evidence of pericardial constriction was noted.

## Discussion

The above report describes a rare case of primary meningococcal pericarditis secondary to serogroup B meningococcal infection in a young female. Primary meningococcal pericarditis is defined as “purulent, culture-positive but without signs of meningeal or other clinical systemic involvement” [8]. Early differentiation between viral and bacterial pericarditis is vital, as cases can quickly progress to the development of pericardial constriction and cardiac tamponade – which can occur in up to 88% of patients with PMP [9]. Early identification and antibiotic administration are of clear importance in order to achieve favourable outcomes, such as protection against pericardial constriction and avoidance of cardiac surgery. The patient discussed in this report received antibiotics six-days after onset of symptoms, and as is shown, no evidence of pericardial constriction was discovered at an outpatient appointment approximately one month following discharge.

Infections caused by meningococcus remain a significant worldwide public health problem. The bacterium itself is split in to 13 serogroups, with six accounting for most cases of life-threatening disease worldwide (A,B,C,W-135, X and Y). This case represents a report of PMP in which serogroup B was isolated (from both blood and pericardial fluid cultures). Interestingly a large report in 2017 found serogroup B to be associated with younger age groups than its counterparts (median age 5 years, compared with 18 for C, 24 for W and 49 for Y) [10]. As such, cases of PMP secondary to group B are exceedingly rare in the literature for a patient in this age group.

The patient discussed in this report received vaccination against Meningococcus A, C, W-135 and Y four years before this acute presentation. At no point did she receive vaccination against Meningococcus B, the serogroup isolated during this presentation. Routine vaccination against Meningococcus B was introduced in the United Kingdom on 1st September 2015, to be offered routinely to all babies born on or after 1st July 2015 [11]. The Joint Committee on Vaccination and Immunisation (JCVI) noted that between 2002 and 2012 Meningococcus B accounted for around 80 % of all cases of infectious meningococcal disease in England and Wales [12]. Studies assessing the impact of the vaccination have consistently demonstrated a significantly reduced incidence amongst vaccinated children. A 2020 study demonstrating a reduction in the incidence of meningococcal group B disease of 75 % (63 observed cases as compared with 253 expected) [13]. Certainly this case provides an individual account of the how this programme may shield future generations from similar cases to the one described here.

In patients presenting with pericarditis the major risk factors associated with poor prognosis include: high fever (>38 °C); subacute course (symptoms over several days without a clear-cut acute onset); evidence of large pericardial effusion; cardiac tamponade and failure to respond within seven days to non-steroidal anti-inflammatory drugs (NSAIDs) [1]. For this reason, the 2015 European Society of Cardiology (ESC) guidelines on management of pericardial diseases propose that, “any clinical presentation that may suggest an underlying aetiology (e.g., a systemic inflammatory disease) or with at least one predictor of poor prognosis (major or minor risk factors) warrants hospital admission and an aetiology search” [1]. This proposition would again be supported by the clinical course outlined in this report and provides a useful framework to help select those minority of patients in whom a more serious underlying cause is present, and further investigation therefore required. Blood cultures provide vital information, and allow us to complete a thorough aetiological search. Empirical antibiotic administration can cloud the clinical picture, making it harder to identify causative organisms and initiate appropriate treatment early; especially in rare cases such as this. To aid the early administration of appropriate antibiotics, it may be pertinent to recommend a low-threshold for performing blood cultures in patients with pyrexia and pericarditis or pericardial effusion, ideally, before any antibiotics have been administered.

## Conclusion

We report an unusual case of a meningococcal pericardial effusion caused by serogroup B. In addition to scientific interest, this report highlights the fact that not all cases of pericarditis and pericardial effusion in young people are related to viral infection, a point which becomes even more pertinent given the timing of this patient’s presentation with non-specific symptoms during a pandemic. Whilst we must clearly accept the rarity of cases such as the one outlined here, there remains good reason to maintain a degree of suspicion for rarer causes and to consider early blood cultures in patients with pyrexia and pericardial effusions. Simple interventions such as this will help to maximise the chances of early detection of and targeted therapy for a pathogen which can cause significant morbidity and mortality.

## Ethical approval

n/a

## Consent

Written informed consent was obtained from the patient for publication of this case report and accompanying images. A copy of the written consent is available for review by the Editor-in- Chief of this journal on request.

## Funding

This research did not receive any specific grant from funding agencies in the public, commercial, or not-for-profit sectors.

## CRediT authorship contribution statement

**Matthew Green**: conceptualization, obtaining consent, literature search, data collection, writing of manuscript and original draft preparation, editing subsequent drafts, submission process, **Peter Harrison**: editing subsequent drafts, data collection, Writing - review & editing **Anshuman Sengupta**: Principle supervision, reading/editing drafts and providing advice, data collection, **Dominik Schlosshan**: Supervision, advice.

## Competing interests

The authors have no competing interests to declare.

## References

[1] Adler Y., Charron P., Imazio M., Badano L., Barón-Esquivias G., Bogaert J. (2015). 2015 ESC guidelines for the diagnosis and management of pericardial diseases: the task force for the diagnosis and management of pericardial diseases of the european society of cardiology (ESC) endorsed by: the European association for cardio-thoracic surgery (EACTS). Eur Heart J.

[2] Martin M., LeWinter M.D. (2014). Acute pericarditis. N Engl J Med.

[3] Imazio M., Cecchi E., Demichelis B., Ierna S., Demarie D., Ghisio A. (2007). Indicators of poor prognosis of acute pericarditis. Circulation.

[4] Blaser M., Reingold A., Alsever R., Hightower A. (1984). Primary meningococcal pericarditis: a disease of adults associated with serogroup C Neisseria meningitidis. Clin Infect Dis.

[5] Imazio M., Cecchi E., Demichelis B., Ierna S., Demarie D., Ghisio A. (2007). Indicators of poor prognosis of acute pericarditis. Circulation.

[6] Pankuweit S., Ristić A.D., Seferović P.M., Maisch B. (2005). Bacterial pericarditis. Am J Cardiovasc Drugs.

[7] Herrick W. (1918). Meningococcal pericarditis, with report of 12 cases. Med Clin North Am.

[8] Finkelstein Y., Adler Y., Nussinovitch M., Varsano I., Amir J. (1997). A new classification for pericarditis associated with meningococcal infection. Eur J Paediatr.

[9] Baevsky R. (1999). Primary meningococcal pericarditis. Clin Infect Dis.

[10] Whittaker R., Gomes Dias J., Ramliden M., Ködmön C., Economopoulou A., Beer N. (2017). The epidemiology of invasive meningococcal disease in EU/EEA countries, 2004-2014. Vaccine.

[11] Ladhani S.N., Campbell H., Parikh S.R., Saliba V., Borrow R., Ramsay M. (2015). The introduction of the meningococcal B (MenB) vaccine (Bexsero®) into the national infant immunisation programme–new challenges for public health. J Infect.

[12] Joint Committee on Vaccination and Immunisation (JCVI). JCVI position statement on use of Bexsero® meningococcal B vaccine in the UK. 2014. 〈https://www.gov.uk/government/publications/meningococcal-b-vaccine-jcvi-position-statement〉.

[13] Ladhani S.N., Andrews N., Parikh S.R., Campbell H., White J., Edelstein M. (2020). Vaccination of infants with meningococcal group B vaccine (4CMenB) in England. N Engl J Med.

